# Nutritional enhancement in plants – green and greener

**DOI:** 10.1016/j.copbio.2019.12.010

**Published:** 2020-02

**Authors:** Johnathan A Napier, Olga Sayanova

**Affiliations:** Rothamsted Research, Harpenden, Herts AL5 2JQ, UK

## Abstract

The global challenges of ensuring sufficient safe and nutritious food for all are enshrined within the Sustainable Development Goals. As our planet's population continues to grow, and as the impacts of climate change and environmental pollution become more visible to all, new solutions continue to be sought as to how best address these. Transgenic crops specifically focussed on delivering health-beneficial compounds will likely play a role in this, and this review will consider several areas where good progress has been made. In particular, the transition from basic research to commercial product is a journey that more and more projects are embarking on, hopefully leading to the fulfilment of earlier promises as to the potential of genetically modified (GM) plants to deliver improved human nutrition.

## Highlights

• Transgenic plants as green factories for the production of compounds with human health benefits.
• Reduced environmental footprint and improved sustainability via GM plants.
• Translation of basic research into tangible products.

## Graphical abstract

**Figure:**
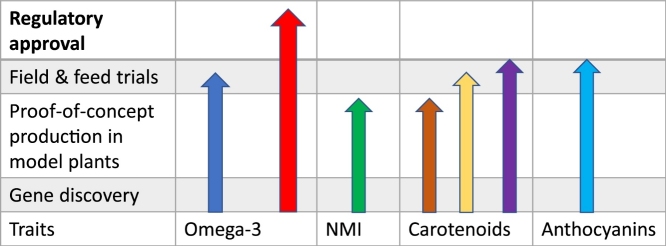

**Current Opinion in Biotechnology** 2020, **61**:122–127

This review comes from a themed issue on **Plant biotechnology**

Edited by **Ralf Reski**, **Ed Rybicki** and **Gary Foster**

For a complete overview see the Issue and the Editorial

Available online 3rd January 2020

**https://doi.org/10.1016/j.copbio.2019.12.010**

0958-1669/© 2019 The Author(s). Published by Elsevier Ltd. This is an open access article under the CC BY license (http://creativecommons.org/licenses/by/4.0/).

## Introduction

The potential of plant genetic engineering to enhance the nutritional composition of plants has been appreciated since the dawn of the era of transgenesis in the early 1980s, but very limited progress has been made in bringing such traits to the market. This is in stark contrast with the so-called input traits (such as herbicide tolerance and insect) which comprise the vast majority (99.9%) of all GM crops grown on the planet today [1^••^]. The reasons for this disparity are many and varied, including the cost of regulatory approval, concerns over consumer acceptance and the more complex nature of the nutritional enhancement traits, but recently progress has been made [1^••^]. Moreover, the increased focus on the bioeconomy and the wider ‘greening’ of society (meaning a more sustainable, less environmentally intrusive approach) has resulted in some philosophical shifts when it comes to using new technologies to provide alternative sources of important nutrients we take for granted [2^•^]. This short review article will consider some exemplars for how plant biotechnology can contribute to improved human health and reduced environmental impact.

### Omega-3 fish oils

**Figure 1:**
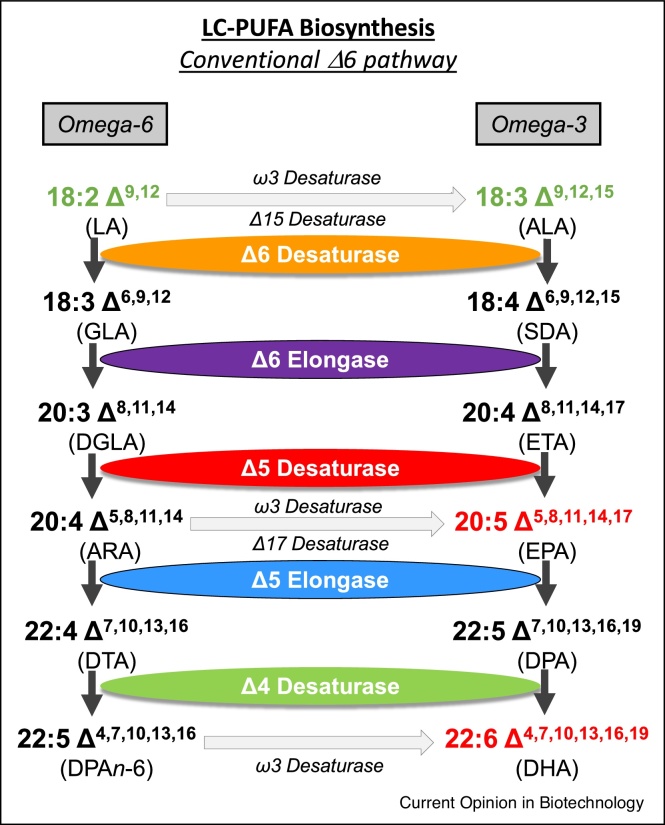
Schematic representation of the biosynthetic pathway for omega-3 LC-PUFAs in transgenic plants. Precursor fatty acids LA and ALA are shown in green. A minimum of three to five enzymatic steps are required to convert these C18 fatty acids to the desired C20 omega-3 EPA or C22 omega-3 DHA (red), Enzymes will recognise both omega-6 and omega-3 substrates, hence the requirement for w3-desaturases to convert omega-6 products to the omega-3 form (e.g. ARA to EPA). This represents a ‘salvage’ pathway for further generation of desired omega-3 LC-PUFAs.

The health benefits associated with a diet containing omega-3 fish oils (or specifically omega-3 long chain polyunsaturated fatty acids) are well-established and are exemplified as nutrients we should consume more over. This is because they contribute to reduced risk of cardiovascular disease (CVD) as well as positive anti-inflammatory lipid mediators and roles in brain and retinal function. Current sources of these omega-3 LC-PUFAs such as eicosapentaenoic acid (EPA) and docosahexaenoic acid (DHA) are almost exclusively derived from our oceans via wild capture of marine fish. This has an obvious implication for the sustainability of such methods as well as consequences for marine pollution and industrial footprint. In addition, as a sad reflection on the state of our oceans, fish accumulate the pollutants present in the water, with the potential to introduce compounds such heavy metals, into the human food chain. For these reasons, considerable effort has been directed to making non-marine sources of omega-3 LC-PUFAs [3], including transgenic plants, predominately using the aerobic desaturase/elongase pathway present in marine microbes (Fig. 1). The past decade has seen significant progress in moving from ‘proof-of-concept’ studies showing the moderate accumulation of EPA and DHA in model systems [4,5] to the successful transfer of the technology to bona fide crops such as canola and camelina [6, 7, 8]. Excitingly, in more recent years, studies have moved beyond the laboratory (‘research’) phase and into a potentially more impactful ‘development’ phase, meaning that these projects are now poised to deliver tangible benefits from the progress. These transitions include carrying out GM field trials (i.e. environmental releases) to provide data on the agronomic performance of the transgenic crop under real-world conditions [9,10] and also feeding studies to confirm the equivalence and efficacy of plant-derived ‘fish oils’ compared with bona fide marine oils. Such studies include feeding trials with mice [11], humans [12^•^] and a number of fish species such as salmon [13,14^•^,^15^] and sea bream [16]. The reason for the strong focus on fish-feeding studies is that the majority of oceanically sourced fish oils are used in aquaculture, again highlighting the sustainability aspect of these projects [3]. Without new *de novo* sources of omega-3 fish oils, the growth of aquaculture industry is likely to be constrained, which is problematic given the growth of the global population and the demand for animal protein [3]. Farmed fish represents the most efficient (in terms of inputs versus edible outputs) production system by which to generate animal protein for human consumption, but the cultivation of marine fish species requires the presence of omega-3 fish oils in the diet, otherwise the animals are unhealthy and lack the very nutrients which make them attractive to the health-conscious consumer. The last ten years has seen a worrying decline in the levels of EPA and DHA in farmed fish [17^•^], due to pressures on availability of oceanic fish oils. Hence, new sources of omega-3 fish oils are keenly sought.

In that respect, the further progress of GM canola lines engineered to accumulate EPA and DHA is important. In two independent efforts [reviewed in Ref. 18^•^], transgenic canola plants making these omega-3 fish oils have successfully been taken through the US regulatory approval system, meaning that these plants can now be grown commercially. This represents the first examples of GM plants with nutritional enhancement traits progressing from research concept to deregulated commercial product, and hopefully marks the start of further examples in the future. Given the demand for new sustainable, clean sources of omega-3 fish oils from the aquaculture industry, it is likely that these plant oils are used predominantly in fish feed [3], but it is equally possible to imagine them being used for direct human nutrition [2^•^,12^•^].

## Non-methylene-interrupted fatty acids and cannabinoid mimetics

**Figure 2:**
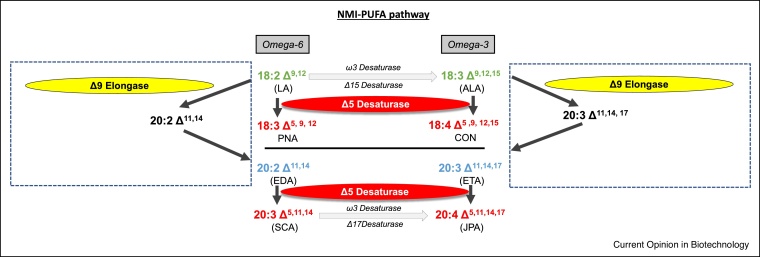
Schematic representation of the biosynthetic pathway for non-methylene-interrupted PUFAs in plants. The ubiquitous essential fatty acids linoleic acid and a-linolenic acid can undergo either Δ5-desaturation to yield the C18 NMI-PUFAs pinolenic acid (PNA; n-6) or coniferonic acid (CON; n-3), or undergo C2 Δ9-elongation to generate a methylene-interrupted intermediate eicosadienoic acid (EDA; n-6) or eicosatrienoic acid (ETA; n-3) which then undergo Δ5-desaturation to yield the NMI-PUFAs sciadonic acid (SCA; n-6) or juniperonic acid (JPA; n-3). The same enzymes (Δ9-elongase, Δ5-desaturase) recognise both omega-6 and omega-3 substrates.

An emerging area of interest, closely related to the LC-PUFAs described above, are non-methylene-interrupted (NMI) fatty acids. These are polyunsaturated fatty acids where their arrangement of double bonds does not follow the usual pattern of occurring every third carbon, separated by a methylene group. Examples of such NMI-PUFAs include the C20 sciadonic acid (SCA;20:3Δ^5,11,14^) and juniperonic acid (JPA;20:4Δ^5,11,14,17^) and C18 pinolenic acid (PNA; 18:3Δ^5,9,11^) [19]. In the case of SCA there is evidence that this fatty acid can displace the omega-6 arachidonic acid (ARA;20:4Δ^5,8,11,14^) from pro-inflammatory cascades and that 2-sciadonoylglycerol (i.e. a monoglyceride with SCA at the sn-2 position) is a ligand for the human CB1 cannabinoid receptor. This has led to the classification of such NMI-PUFA-containing lipids as endocannabinoids [20] with cannabimimetic properties [21]. Given the growing interest in non-psychotropic cannabinoids for a range of human uses, new sources of such lipids are sought (Figure 1, Figure 2). One of the enzymatic activities involved in the biosynthesis of SCA, NMI-PUFA Δ5-desaturase has been identified in seeds of *Anemone leveillei*, a member of an angiosperm family of *Ranunculaceae* and co-expression of this desaturase and a Δ9-elongase in transgenic Arabidopsis resulted in accumulation of SCA and JPA [19]. Very recently, de novo transcriptome sequencing of seed RNA from the gymnosperm *Torreya grandis* (Chinese yew tree) has identified a number of potential genes involved in the biosynthesis of SCA [22]. Further works remains to be done to confirm the function of these *T. grandis* putative desaturases and elongases and the configuration of the PUFA-biosynthetic pathway they represent, but such advances may provide a molecular toolkit for the transgenic synthesis of NMI-PUFAs, analogous to previously demonstrated for the omega-3 fish oils [23^•^].

## Astaxanthin and related carotenoids

The ketocarotenoid astaxanthin is used in a number of different feed and food applications, predominantly as a colourant in aquafeed diets but also as an antioxidant and nutraceutical with anti-inflammatory properties. Natural sources of astaxanthin include a diverse range of bacteria and marine microalgae, and its characteristic red-pink colour is present throughout many aquatic foodwebs (examples include the pigmentation present in salmonid species and krill), indicating amenability to accumulation via dietary intake [24]. Despite the presence of different biological sources, the majority of commercial astaxanthin is chemically synthesised – however, this usually generates a mixture of three different optical isomers, which has reduced capacity as an antioxidant. Perhaps more importantly, the isomerically homogenous natural astaxanthin is considered more efficacious in nutraceutical applications and human health [24]. Previous attempts to engineer transgenic plants to synthesis astaxanthin have met with some success, focussing on the expression of heterologous enzymes to convert the endogenous carotenoid β-carotene. This has predominantly (but not exclusively) used bacterial activities such as β-carotene hydroxylase (*CrtZ*) and oxyxgenase (*CrtW*), targeted to the plastid [24]. Proof-of-concept accumulation of astaxanthin and related ketocarotenoids has been demonstrated in a number of different plant species, though the overall yield is not currently sufficient to compete with other sources [25]. Recent studies have indicated the potential of GM plants to provide a ‘green’ source of this pigment for use in aquaculture, with transgenic tomato fruit engineered to accumulate astaxanthin which was subsequently used to formulate diets for aquafeed studies on trout [26^••^]. Interestingly, much of the ketocarotenoid generated in this study was not astaxanthin but instead the structurally related phoenicoxanthin and canthaxanthin. Moreover, these ketocarotenoids were predominantly esterifed with fatty acids, as opposed to the free form. Irrespective of that, when evaluated as an aquafeed ingredient, these compounds performed equivalent to astaxanthin in terms of the pigmentation of the flesh [26^••^], indicating that at least for some applications, the purity and form of the ketocarotenoid is not critical. Astaxanthin for use in aquaculture has also been made in GM soybeans, stacked with an omega-3 trait [27]. Since these two ingredients are the most expensive components of aquafeed diets, the possibility to make them both in one platform represents an exciting development. Feeding trials were carried out with trout and kampachi, allowing for an evaluation of diets with reduced marine-derived ingredients but improved sustainability criteria [27].

Further technical refinements have been made to the transgenic approaches used to synthesised astaxanthin. For example, an artificial glycine-rich polypetide linker was used to fuse together bacterial *CrtW* and *CrtZ* sequences, with the goal of increasing the flux of substrate through to astaxanthin [28^•^]. This approach was shown to be successful in both prokaryotic and eukaryotic systems, enhancing the accumulation of astaxanthin and decreasing phoenicoxanthin and canthaxanthin [28^•^], and may represent a simple method by which to seed the assembly of a metabolon.

Probably the best-known example of engineering the accumulation of carotenoids in GM plants is Golden Rice, which also represents one of the first (and still incomplete, in terms of regulatory approval) examples of transgenic nutritional enhancement [1^••^]. A recent systematic re-examination of the metabolic engineering of rice endosperm for the accumulation of β-carotene confirmed the importance of generating sufficient flux through the methylerythritol 4-phosphate (MEP) pathway [29] and also presented a new platform for the synthesis of astaxanthin and other ketocarotenoids in a stable food crop. Finally, in a very elegant and distinct approach, transplastomic (as opposed to nuclear transformation) was used to engineer tobacco plants with the capacity to synthesise astaxanthin [30^••^]. This took advantage of the plastidial location of the MEP pathway which generates the isoprenoid precursors for carotenoid synthesis and also the prokaryotic nature of this organelle which makes it an obvious host for bacterial *CrtW* and *CrtZ* genes. Stable transformation of the tobacco plastid genome was successfully achieved, and high levels of astaxanthin, but not other ketocarotenoids, was achieved [30^••^]. This plastidial trait was also transferable to related Nicotiana species by grafting, since plastids are known to migrate across grafts [30^••^].

## Anthocyanins

Anthocyanins comprise a large family of water-soluble pigments, not only prized for their different hues and colours, but also now recognised as being vital health-protective components of the human diet [31]. They can reduce the risk of CVD and related inflammatory-response pathologies, as well as a number of cancers of the digestive tract [32,33]. Although a wide range of native organisms accumulate different anthocyanins, many are not amenable or appropriate for cultivation and/or harvest. Transgenic plants have been developed as chassis for the synthesis of a designer palette of anthocyanins with considerable success. Unlike the preceding examples of omega-3 fatty acids and ketocarotenoids, accumulation of anthocyanins has been achieved the overexpression of regulatory genes (i.e. transcription factors), in addition to specific biosynthetic genes [34]. Such an approach has allowed for the high-level, tailored synthesis of specific subclasses of anthocyanins, in both transgenic plants [35] and cell suspension cultures [36^•^]. Similar approaches of using transcription factors to upregulate whole classes of beneficial secondary metabolites such as polyphenols have been used successfully [37], not only providing a new sustainable production source but also helping to elucidate the key enzymatic steps in the biosynthesis of these compounds [37]. Certainly, the contribution of transcription factors in the domestication of a number of crops selected for their aroma and appearance is now becoming apparent [38^••^], helping to revealing a fascinating and complex pedigree and the blinded contribution of man and environment [38^••^,^39^]. Given this greater understanding of the endogenous regulatory factors which modulate the synthesis and accumulation of health-beneficial phytochemicals and secondary metabolites, it should be possible to select plants via conventional marker-assisted breeding techniques with superior profiles for better human nutrition [2^•^].

## Conclusions

As described here, significant progress has been made in the last few years to not only demonstrate the feasibility of using transgenic plants as platforms to make a range of health-beneficial compounds, but also to move further down the translation pipeline ultimately towards regulatory approval, commercialisation and consumer uptake. Part of this process involves the transition from a research phase to a development mode [1^••^], which not only takes time and money but also an entirely different mindset and approach. It is genuinely exciting that some of the projects described above are already on that journey, and it is to be hoped that soon there will be tangible products from these efforts. But as recently noted [40] by Marc Van Montagu, one of the ‘founding fathers’ of plant genetic engineering, it will only be possible to capture the truly big and disruptive innovations we require to meet the challenges of the Sustainable Development Goals (SDGs) if we work collaboratively with co-design approaches and common goals endorsed by social license. That, in turn, will likely needs a more thorough considerations of consumer motivation [41] and the ethical frameworks around the use of GM crops, as exemplified in this thoughtful review [42^•^].

## Conflict of interest statement

Nothing declared.

## References and recommended reading

Papers of particular interest, published within the period of review, have been highlighted as:

• of special interest
•• of outstanding interest
